# Cytosolic Peroxiredoxin *TSA1* Influences Acetic Acid Metabolism and pH Homeostasis in Wine Yeasts

**DOI:** 10.1021/acs.jafc.4c13199

**Published:** 2025-03-22

**Authors:** Víctor Garrigós, Cecilia Picazo, Lisa Dengler, Jennifer C. Ewald, Emilia Matallana, Agustín Aranda

**Affiliations:** † Institute for Integrative Systems Biology (I2SysBio), Universitat de València-CSIC, C/Catedrático Agustín Escardino 9, 46980 Paterna, Valencia, Spain; ‡ Interfaculty Institute of Cell Biology (IZB), 9188 University of Tuebingen, Auf der Morgenstelle 15, 72076 Tuebingen, Germany

**Keywords:** Tsa1p, acetic acid, wine yeasts, aldehyde dehydrogenases, wine

## Abstract

Acetic acid is a key metabolite in yeast fermentation, influencing wine quality through its role in volatile acidity. In *Saccharomyces cerevisiae*, acetic acid production involves aldehyde dehydrogenases, primarily Ald6p during fermentation and Ald4p under respiratory conditions. However, the regulatory mechanisms of these enzymes throughout fermentation and how they differ in commonly used strains remain partially unclear. This study explores cytosolic peroxiredoxin Tsa1p as a novel regulator of acetic acid metabolism. *TSA1* gene deletion revealed strain-dependent effects on acetic acid metabolism and tolerance, showing reduced production and enhanced consumption in the laboratory media. Under respiration, Ald4p-driven acetic acid production, which raises extracellular pH, was mitigated by the absence of Tsa1p. During wine fermentation, *TSA1* deletion decreased the initial acetic acid surge by downregulating the *ALD6* transcription and enzymatic activity. These findings establish Tsa1p as a metabolic regulator and a potential target for modulating acetic acid levels to manage volatile acidity and improve wine quality.

## Graphical abstract

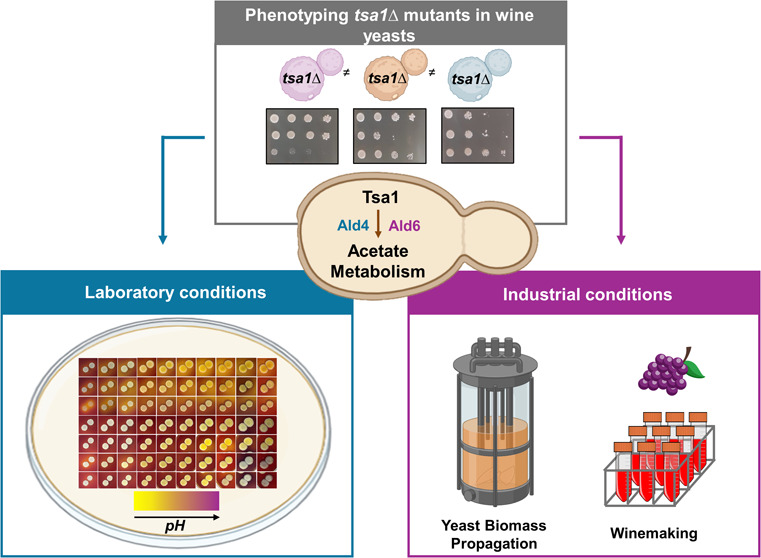

## Introduction

Volatile acidity is a relevant parameter of wine that derives from acetic acid and related compounds, like ethyl acetate, either in their free form or bound as salts, as outlined in the resolution of the OIV-OENO 662C-2022. Its main component is acetic acid, which causes a pungent smell associated with vinegar and wine spoilage.^1^ That perception is dependent on the type of wine and threshold, and the sensory threshold for the detection of volatile acidity is often cited as 0.7 g/L.^2^ The species of yeast *Saccharomyces cerevisiae* can produce acetic acid during grape juice fermentation.^3^ Under respiratory conditions, acetyl-CoA is produced directly from pyruvate by the mitochondrial pyruvate dehydrogenase (PDH) complex. This complex is inactive during grape juice fermentation, so the cytosolic acetyl-CoA required for processes such as lipid synthesis is produced via the PDH bypass pathway (Figure S1).^4^ First, pyruvate from glycolysis is decarboxylated to acetaldehyde by pyruvate decarboxylase (PDC). While most acetaldehyde is reduced to ethanol by alcohol dehydrogenases (ADHs) to maintain the redox balance, a fraction is further oxidized to acetic acid by aldehyde dehydrogenases (ALDHs). Acetic acid is subsequently converted into acetyl-CoA through the action of acetyl-CoA synthases (ACS), a reaction that requires ATP. There are a variety of ALDH activities described in yeast, distributed in two cellular locations.^5^ Cytosolic ALDHs include Ald6p (the major one) and the stress-induced Ald2 and Ald3, while mitochondrial ALDHs are encoded by *ALD4* (the major one) and *ALD5*. Ald6p is dependent on magnesium ions, while Ald4p uses potassium, and the *ALD4* gene is glucose repressed while *ALD6* is fully active during fermentation.^6^ This would explain why, during grape juice fermentation, the role of cytosolic isozyme Ald6p in the production of acetic acid is more relevant than its mitochondrial counterpart Ald4p.^7^

Acetic acid production in *S. cerevisiae* depends on many environmental factors, including the nitrogen, vitamin, and lipid composition of the grape juice, the initial sugar concentration, and physical parameters like temperature.^3,8–11^ Oxygen plays a pivotal role in its production, as well. While aeration has been a tool to reduce ethanol content, it can activate respiratory pathways, leading to an undesirable increase in volatile acidity.^12^ Ethanol reduction is a relevant challenge in modern enology, as rising sugar levels in grapes due to global warming result in elevated alcohol levels in wine.^13–15^ Efforts to divert glycolytic flux toward glycerol production by genetic manipulation have shown the potential for reducing ethanol levels. However, these modifications also lead to redox imbalances, leading to an unpleasant rise in acetic acid production that requires additional deletion of ALDH genes.^16^ Therefore, the regulatory mechanisms governing acetic acid metabolism are of great interest to winemaking researchers to tackle the dual challenges of managing alcohol content and volatile acidity.^17^

Previous studies from our laboratory have demonstrated that redox signaling influences metabolic processes under winemaking conditions.^18–20^ One of the main redox systems responsible for oxidative damage repair and cellular redox control is the cytosolic peroxiredoxin–thioredoxin–thioredoxin reductase system.^21^ At the core of this system is Tsa1p, the principal cytosolic peroxiredoxin, which detoxifies a range of peroxides, including hydrogen peroxide (H_2_O_2_). The catalytic mechanism of Tsa1p involves the formation of a disulfide bond between its peroxidatic cysteine (*C*_P_) and the resolving cysteine (*C*_R_) during peroxide reduction. The disulfide bond is subsequently reduced by the cytosolic thioredoxins Trx1/2p, which are regenerated by thioredoxin reductase (Trr1p) using NADPH as an electron donor. Beyond its role in peroxide detoxification, Tsa1p also acts as a chaperone^22^ and as a regulator of the cAMP-protein kinase A (PKA) signaling pathway through direct redox control of its catalytic subunits, linking redox regulation to cellular metabolism and aging.^23^ Hence, this redox system plays a crucial role in repairing oxidative damage in key cysteines across a variety of enzymes,^24–26^ further highlighting its potential involvement in metabolic regulation. For instance, *TRR1* deletion disrupts the TCA cycle and leads to an increase in proteogenic amino acids.^20^ The *trx1*Δ*trx*2Δ double mutant shows diminished glycolytic activity while enhancing lipid synthesis and amino acid metabolism during wine fermentation.^19^ Additionally, *TSA1* deletion decreases acetic acid production during grape juice fermentation and enhances trehalose accumulation at the end of biomass propagation in molasses.^18^

This work provides a detailed examination of the role of Tsa1p in acetic acid metabolism. *TSA1* and *TRR1* deletion alters acetic acid production and consumption in a manner that varies across different growth conditions and media. In addition, genetic diversity among commercial wine strains contributes to differential tolerance to acetic acid. Cytosolic magnesium-dependent ALDH activity is controlled by Tsa1p during the initial hours of grape juice fermentation. However, mitochondrial potassium-dependent ALDH activity is also influenced by the absence of the *TSA1* gene under low-glucose respiratory conditions. Therefore, these findings highlight the complexity of acetic acid metabolism regulation through the redox-controlling peroxiredoxin–thioredoxin system.

## Materials and Methods

### Yeast Strains, Growth Media, and Conditions

The yeast strains used herein are listed in Table S1. Deletion mutants were generated in the laboratory strain W303 and the haploid wine strain C9 ^27^ using the reusable kanMX marker. This marker was amplified via PCR from the pUG6 plasmid^28^ and contains loxP sequences for excision by the Cre recombinase from the YEp-cre-cyh plasmid.^29^ In the industrial wine strains L2056, T73, and EC1118, deletions of the *TSA1* and *TRR1* genes were performed by CRISPR-Cas9 using the pRCC-K plasmid, a gift from Eckhard Boles (Addgene plasmid #81191), and following the protocol described.^30^ Yeast transformations were carried out using the lithium acetate method.^31^

For maintenance and standard propagation purposes, yeast cultures were grown at 30 °C in rich YPD medium (1% yeast extract, 2% bactopeptone, and 2% glucose). Solid media were prepared by supplementing with 2% agar, and Geneticin at a concentration of 200 mg/L was used for selecting the kanMX transformants. To evaluate the phenotypic traits of the strains under alternative standard laboratory conditions, cells were cultivated in a glucose minimal medium (GMM) as previously described.^32^ Minimal medium SD contained 0.17% yeast nitrogen base, 0.5% ammonium sulfate, and 2% glucose. Media was purchased from Condalab (Spain), and Geneticin from Duchefa Biochemie (Netherlands).

For growth spot analysis, serial dilutions were prepared from stationary-phase cultures grown in YPD, and 5 μL drops were placed onto selective media. When required, the carbon source in YPD was substituted with 2% fructose (YPF), sucrose (YPS), glycerol (YPG), or ammonium acetate (YPA). For stress analysis, the SD medium was used by adding 0.17% acetic acid or 10% ethanol.

To investigate the impact of yeast colony growth on extracellular pH, a solid growth medium was prepared as described.^33^ Extracellular pH was monitored using the “giant colony” growth method.^34^ An overnight culture grown in YPD was diluted to an OD (600 nm) of 0.01, and 10 μL were spotted onto plates and incubated at 30 °C. To quantitatively investigate the impact of metabolic transitions on extracellular pH, yeast cells were cultured under low-glucose conditions. Cultures were inoculated at an initial OD (600 nm) of 0.05 and incubated at 30 °C with shaking at 220 rpm for 24 h. The liquid medium contained 1% yeast extract and 0.2% glucose, with the pH adjusted to 5.5 using HCl. Extracellular pH was determined using a colorimetric assay employing the pH-sensitive dye BCP (Merck, Germany).^33^

Bioreactor-scale assays were conducted using synthetic molasses (SM), prepared as previously described,^18^ in a 5 L ez2-Control bioreactor (Applikon Biotechnology, Netherlands) equipped with proportional, integral, and derivative (PID) control units for pH, temperature, oxygen, and agitation speed. The bioreactor containing 3 L of sterilized SM was inoculated with an initial OD (600 nm) of 0.1 from YPD precultures.

Wine fermentation experiments were performed using synthetic must (MS300) prepared as described.^35^ It contains glucose and fructose at 10% each, malic 3 g/L, citric 0.3 g/L, and tartaric acid 3 g/L, nitrogen source 300 mg N/L (120 mg as (NH_4_)Cl and 180 mg as amino acids), mineral salts (KH_2_PO_4_ 750 mg/L, K_2_SO_4_ 500 mg/L, MgSO_4_ 250 mg/L, CaCl_2_ 155 mg/L, and NaCl 200 mg/L), oligoelements, vitamins, ergosterol 15 mg/L, and oleic acid 5 mg/L, at pH 3.3. Cells from 2 day YPD cultures were inoculated into 30 mL of must in conical centrifuge tubes at a final concentration of 10^6^ cells/mL, and the cultures were incubated at 25 °C with low agitation (50 rpm). The vinification progress was monitored by measuring sugar consumption using the dinitro-3,5-salicylic acid (DNS, Merk, Germany) according to Miller’s method.^36^

### Metabolite Measurements

Acetic acid and ammonia concentrations were determined spectrophotometrically using coupled enzymatic reactions linked to NAD^+^/NADH and NADP^+^/NADPH redox pairs. Commercial kits from Megazyme Ltd. (Bray, Ireland, www.megazyme.com; K-ACET and K-AMIAR kits) were employed for these measurements, following the manufacturer’s protocols. high-performance liquid chromatography (HPLC) was employed to quantify acetic acid in the GMM samples. HPLC analysis was performed on a Shimadzu HPLC system equipped with a Repromer H column from Maisch GmbH, Germany. A sample volume of 10 μL was injected onto the column using an autosampler cooled to 4 °C. Metabolites were eluted isocratically with 5 mM H_2_SO_4_ (sulfuric acid solution, 49 to 51%, For HPLC, Honeywell Fluka). Samples were run with a flow rate of 1.0 mL/min for 25 min at 40 °C. The analytes were monitored using a refractive index detector (Shimadzu RID-20A).

### Measurement of Enzymatic Activities

Cytosolic and mitochondrial aldehyde dehydrogenase activities were measured using cell extracts from cultures grown under the winemaking conditions described above. Cell extracts were prepared by washing and resuspending yeast cells in 0.5 mL of a buffer containing 10 mM sodium phosphate (pH 7.0), 1 mM dithiothreitol, and 5% (w/v) glycerol. Cell disruption was achieved with glass beads using a Precellys Evolution homogenizer (Bertin Technologies, France), operated for three 20 s intervals at a speed of 4.5 m/s, with 1 min cooling periods between each interval. Protein concentration was measured using the DC Protein Assay (BioRad), following the manufacturer’s protocol. Cytosolic and mitochondrial aldehyde dehydrogenase activities were assessed.^6^ In both cases, the reaction was initiated by the addition of acetaldehyde, and the increase in absorbance at 340 nm was monitored by using a Varioskan LUX microplate reader (Thermo Fisher Scientific).

### Relative Gene Expression Level Quantification by Real-Time PCR and Western Blot

To quantify the relative expression levels of the target genes, yeast cells were harvested at the specified time points, and total RNA was extracted as described previously.^37^ The extracted RNA was reverse transcribed using the NZY First-Strand cDNA Synthesis Kit (Nzytech, Portugal). Gene-specific primers^38^ were used to amplify *ALD4* and A*LD6*. The housekeeping gene *ACT1* served as an internal control. Quantitative PCR (qPCR) was performed using the NZYSpeedy qPCR Green Master Mix (Nzytech, Portugal) on a QuantStudio 3 instrument (Thermo Fisher Scientific), following the manufacturer’s protocol. Each reaction was conducted in triplicate, and the average cycle threshold (Ct) value from triplicate samples was used for analysis. Relative transcript levels were calculated by using the 2^–ΔΔCt^ method. For Western blot, cells were lysed using glass beads, and whole-cell extracts were prepared in lysis buffer containing 1 M Tris-HCl (pH 7.5), 5 M NaCl, 1 M MgCl_2_, 10% (v/v) NP40, 0.1 M PMSF, and a commercial protease inhibitor tablet (complete Mini, EDTA-free; Roche). Proteins were resolved in an sodium dodecyl sulfate–polyacrylamide gel electrophoresis (SDS–PAGE) gel using an Invitrogen mini-gel system and transferred to poly­(vinylidene difluoride) (PVDF) membranes using a Novex semidry transfer system (Invitrogen, Carlsbad, CA). Immunodetection was performed with the anti-2-Cys-Prx (Abcam) or anti-Prx-SO_3_ (Abcam) antibodies. Anti-Pgk1 (Invitrogen) was used as a loading control. Signal detection was carried out using the ECL Western Blotting Detection System (GE Healthcare) following the manufacturer’s instructions.

## Results

### *TSA1* Deletion Has an Impact on the Metabolism of Wine Yeasts

Previous results indicated that the deletion of peroxiredoxin *TSA1* and heterozygous deletion of one of the copies of thioredoxin reductase *TRR1* reduced the amount of acetic acid during winemaking.^15^ To determine whether this mutation influences acetic acid production in a completely different and industrially relevant environment, the same mutants were tested during growth in synthetic molasses in a bioreactor (Figure 1). This medium mimics the composition of industrial molasses used for yeast biomass propagation, albeit with a reduced sucrose concentration, to facilitate early investigation of the transition to respiratory metabolism. As previously described, under these conditions,^18^ although the *tsa1*Δ mutant cells reached saturation after 24 h of growth, they were unable to achieve the same cell density as the parental strain and remained so even during the fed-batch phase. The *trr1*Δ/*TRR1* mutant showed a similar growth profile to the wild-type strain.^18^ During the growth in batch, at 6 h, the amount of acetic acid was low, below 0.1 g/L, and slightly reduced in the mutants. Acetic acid accumulates to reach a maximum at 24 h in all strains. At this point, the acetate levels in both mutants are comparable to those observed in the wild-type strain. After 24 h, sucrose is fully consumed (data not shown), and acetic acid decreases in the wild-type strain, reaching a minimum at 51 h. In contrast, acetic acid levels in the *tsa1*Δ mutant remain elevated during this period. In the *trr1*Δ/*TRR1* mutant, the acetic acid reduction is delayed and incomplete compared with that of the wild type. At 51 h, fed-batch growth was initiated to sustain yeast respiratory metabolism. During this final stage of biomass production, acetic acid levels increase in both the wild type and the *trr1*Δ*/TRR1* mutant, although this starts earlier in this mutant. Notably, the levels of the *tsa1*Δ mutant remain high throughout the process. These findings indicate that Tsa1p has a relevant impact on acetic acid metabolism during sucrose fermentation, with an effect that appears to differ from its role during microvinification, potentially acting in the opposite direction.

**Figure 1:**
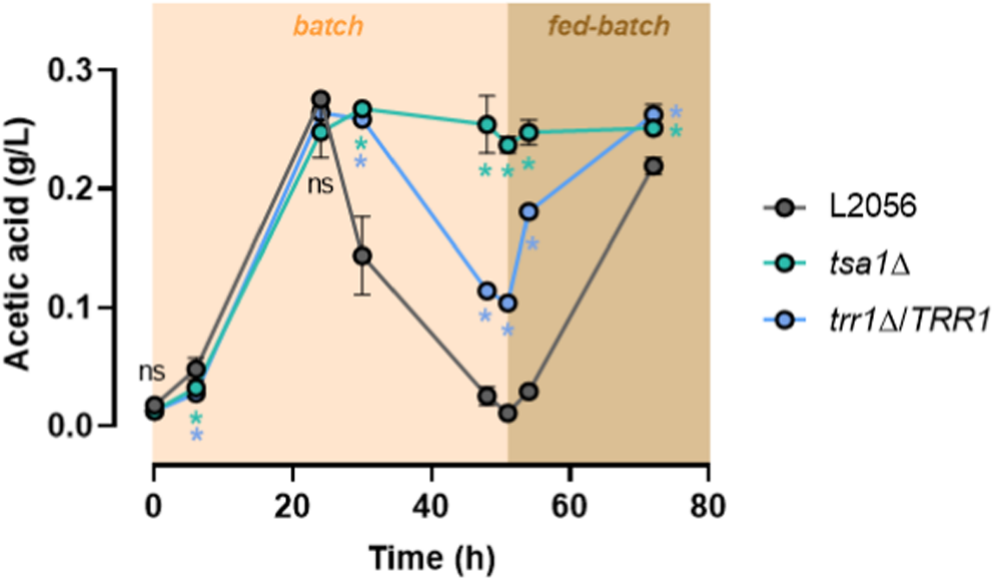
*TSA1* deletion increases acetic acid levels during molasses fermentation. L2056, L2056 *tsa1*Δ, and L2056 *trr1*Δ*/TRR1* strains were grown in the synthetic molasses medium with 2% sucrose in a bioreactor in batch (up to 51 h) and fed-batch conditions. Acetic acid was measured by triplicate, and the mean and standard deviation are shown. Significant differences (*p < 0.05, Student’s *t-*test) between the mutants and the wild-type strain are shown.

As volatile acidity is a relevant phenotype during winemaking and its production varies among commercial wine strains, our next goal was to determine the comparative impact of *TSA1* and *TRR1* mutations on carbon metabolism and stress tolerance across several commercial wine yeasts. To this end, we developed a CRISPR-Cas9 method capable of efficiently deleting all copies of *TSA1* and *TRR1* simultaneously, addressing the diploid nature of most commercial strains. In addition to L2056, we included EC1118, a widely used commercial strain, and T73, another reference strain used in our laboratory. To test the growth of these strains and their corresponding *TSA1* and *TRR1* mutants, spot assays were performed on different substrates (Figure 2A). In the standard rich glucose-containing medium YPD, *TSA1* deletion does not impact growth. As expected,^20^ *TRR1* deletion did, particularly in the L2056 genetic background. This may explain why the homozygous mutant was not obtained in the L2056 strain using the classical homologous recombination approach, and a heterozygous mutant was used.^18^ Similar results were observed when using other easy-to-assimilate hexoses such as fructose (YPF) and a disaccharide such as sucrose (YPS), the latter commonly used in yeast biomass propagation. Therefore, *TSA1* deletion does not impact the growth on fermentable sugars. When a nonfermentable, respiratory carbon source such as glycerol was tested, a similar pattern was observed: *tsa1*Δ mutants displayed no additional phenotype changes. This suggests that *TSA1* deletion does not broadly affect either fermentable or respiratory metabolism.

**Figure 2:**
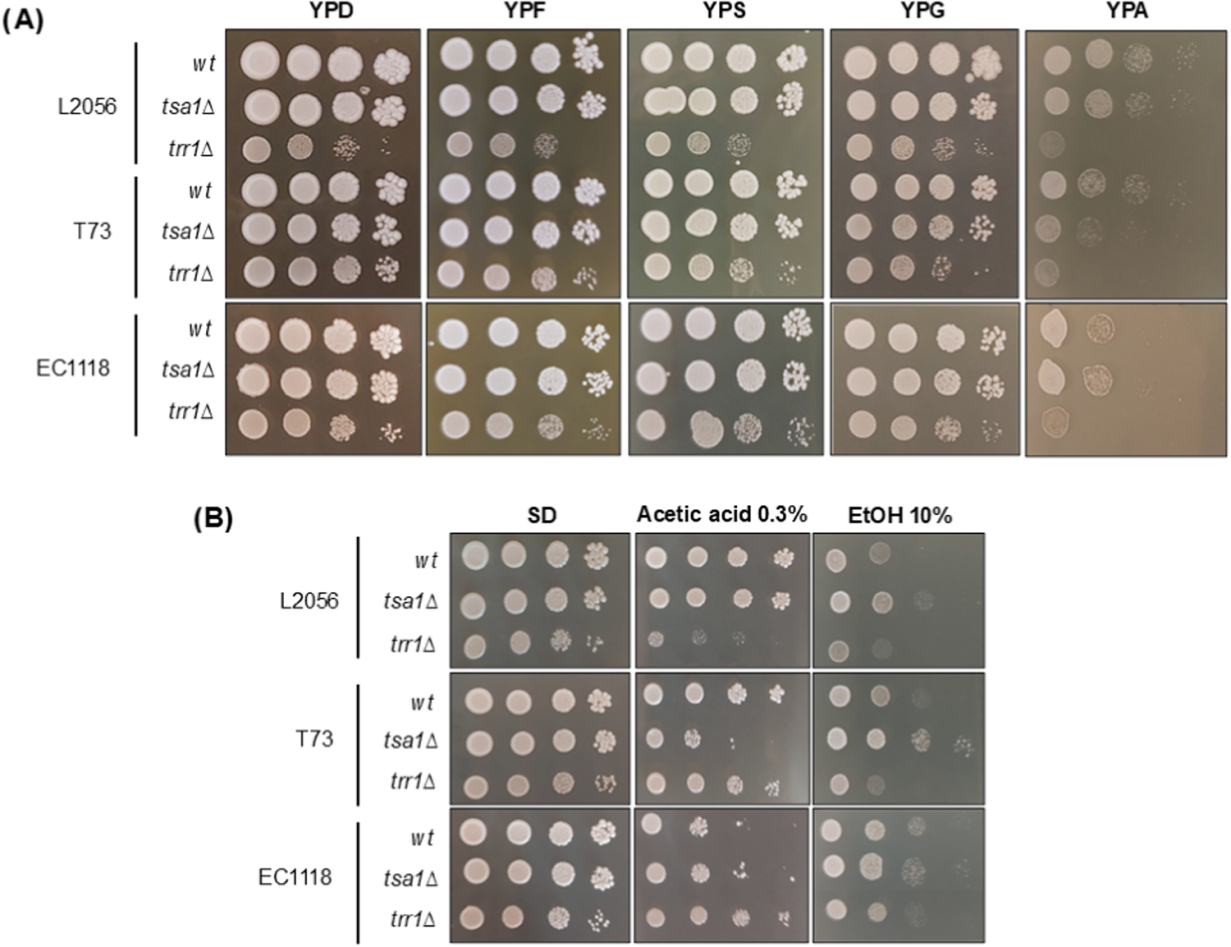
Mutations in *TSA1* and *TRR1* show phenotypic dependence on genetic background. CRISPR-Cas9 deletions of *TSA1* and *TRR1* genes in the commercial wine strains L2056, T73, and EC1118 were tested in different conditions by drop assay. Stationary cultures in YPD were normalized according to OD_600_, serial dilutions were performed, and 5 mL drops were placed in different media. (A) Growth in plates of rich media with different carbon sources: 2% glucose (YPD), fructose (YPF), sucrose (YPS), glycerol (YPG), and ammonium acetate (YPA) were used. (B) Stress caused by 0.3% acetic acid and 10% ethanol in minimal medium SD plates was assessed.

However, when acetate was used as the sole carbon source, growth was generally delayed, particularly for the *TRR1* deletion mutants. Interestingly, while *tsa1*Δ mutants in L2056 and EC1118 strains behaved similarly to their parental strains, T73 *tsa1*Δ *s*howed delayed growth, indicating that *TSA1* plays a more significant role in acetate assimilation in this genetic background. Next, the impact of a stressful amount of acetic acid was tested (Figure 2B).^39^ This experiment is usually conducted in a glucose-containing minimal medium SD, where *TSA1* deletion had a negligible impact on growth. When acetic acid was applied, *TSA*1 deletion negatively affected growth in the T73 background but not in the L2056 and EC1118 strains. Interestingly, *TRR1* deletion exhibited a stronger negative impact in the L2056 background compared to the other strains. A different stress was applied to rule out general stress sensitivity. High ethanol (10%), a relevant stress during winemaking, was tested. Under these conditions, *TSA1* deletion increased tolerance to ethanol, with a mild effect in L2056 and a slightly greater effect in T73, while no impact was detected in EC1118. In contrast, the *TRR1* deletion mutant displayed slightly increased ethanol sensitivity in T73 but not in the other strains. These findings highlight the influence of genetic background on the phenotypic outcomes of deleting components of the peroxiredoxin–thioredoxin system under adverse conditions. Since T73 exhibited the most pronounced responses, it was selected for subsequent experiments.

### Tsa1p Plays a Role in Acetic Acid Production and Consumption during Growth

To assess the impact of *TSA1* deletion on acetic acid metabolism during growth, T73 and T73 *tsa1*Δ strains were grown in a glucose minimal medium (GMM), enabling the monitoring of acetic acid dynamics across metabolic phases under laboratory standard conditions. Both strains exhibited comparable glucose uptake (Figure 3A) and ethanol production (Figure 3B) rates. While the absolute values for glucose consumption and ethanol production suggest a minor influence of Tsa1p on fermentative metabolism under these conditions, the overall yield remains unaffected. This is likely due to the slight growth defect observed in the *tsa1*Δ mutant strain (Table S2). This indicates that Tsa1p has a slight influence on the central carbon metabolism under these particular conditions.

**Figure 3:**
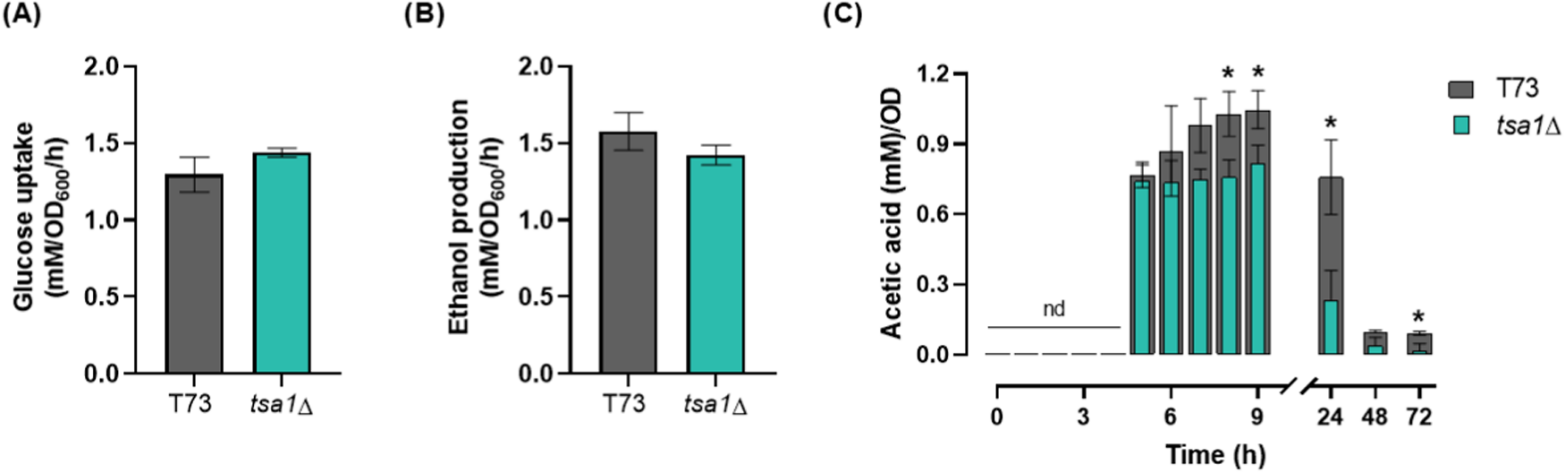
Tsa1p impacts acetic acid production and consumption in a laboratory growth medium in a wine yeast strain. (A) Glucose uptake, (B) ethanol production, and (C) acetic acid yields in GMM medium by T73 and T73 *tsa1*Δ strains. Experiments were carried out in triplicate, and the average and standard deviation are provided. Significant differences (*p < 0.05, Student’s *t*-test) between the mutants and their parental strains are shown. nd, not detected.

In contrast, the acetic acid metabolism showed greater differences (Figure 3C). In the T73 strain, acetic acid was produced during growth and slightly increased, reaching a maximum at the diauxic point. After that, its concentration decreased to very low levels when ethanol was fully consumed. Therefore, respiratory metabolism also uses acetic acid as a nutrient. The mutant strain exhibited a similar overall profile, but absolute acetate levels were lower after 8 h of growth, the maximum concentration was also reduced, and its decline was faster, particularly at 24 h. At the final point of the experiment (72 h), these differences also remained significant. Therefore, Tsa1p seems to be involved in both acetic acid production and consumption in wine yeasts under laboratory conditions. In order to determine whether this effect is conserved in a laboratory strain, the same experiment was carried out in the W303 genetic background (Figure S2). The glucose uptake and ethanol production rates were similar, with no differences between the wild type and the *tsa1*Δ strains. The acetic acid yield profile in the *tsa1*Δ mutant was nearly identical to that of its parental strain, with no significant differences. These findings underscore the phenotypic differences between industrial and laboratory strains, emphasizing the importance of validating laboratory-derived claims in industrial wine strains. Based on these observations under laboratory conditions, we next aimed to investigate whether Tsa1p really does not influence the acetate-driven extracellular pH homeostasis in wine strains, as previously reported.^34^

### Media Acidification is Impacted by Peroxiredoxin Mutation

It has been described that during growth in solid media, yeast colonies acidify their surroundings through acetic acid excretion.^34^ This phenomenon can be followed using a pH-sensitive dye, bromocresol purple, incorporated into the growth medium (Figure 4A). Drops of cultures from T73, *tsa1*Δ, and *trr1*Δ strains were placed on YPD plates buffered at pH 6.5, and the acidification around the “giant colonies” was followed over 2 weeks. In the wild-type strain, the yellow halos indicating acidification started around day 8, persisted until day 10, and then the medium was realkalized by day 13. In the *tsa1*Δ mutant, the acid burst was delayed to day 10 but similarly realkalized by day 13.

**Figure 4:**
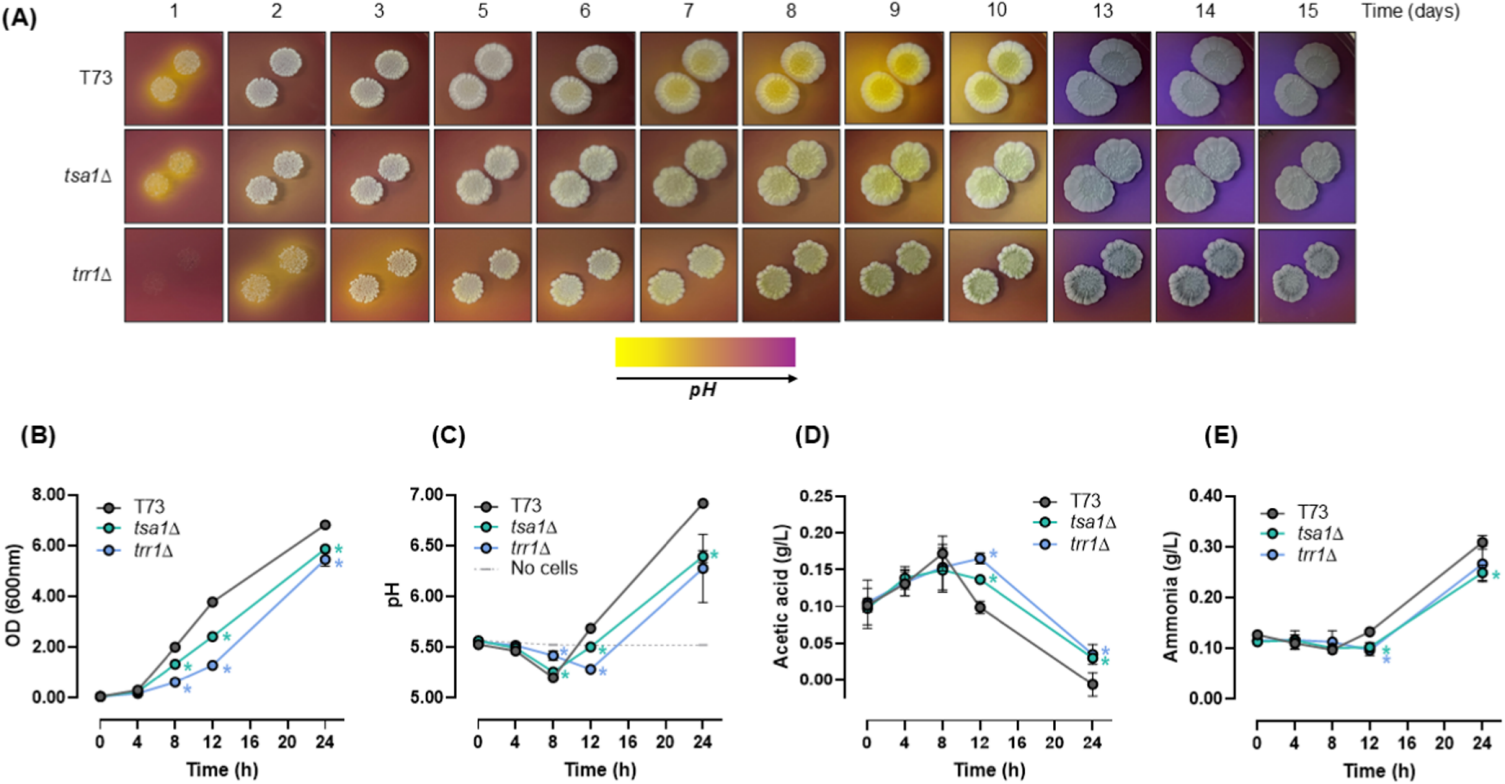
Deletion of *TSA1* reduces extracellular pH by maintaining higher acetic acid levels. The impact of *TSA1* deletion on external pH was measured with T73, T73 *tsa1*Δ, and T73 *trr1*Δ *s*trains in two media, YPD plates buffered at pH 6.5 with 0.01% bromocresol purple, BCP (A) and a liquid medium with 0.2% glucose as carbon source, pH 5.5 (B–E). (A) Drops of both strains were placed on plates with BCP to obtain “giant colonies”, and changes in external pH were monitored for 15 days. (B) Growth measured as optical density (OD) at 600 nm. (C) Extracellular pH. (D) Acetic acid concentration. (E) Ammonia concentration. Experiments in liquid medium (B–E) were carried out in triplicate, and the average and standard deviation are provided. Significant differences (*p < 0.05, Student’s *t-*test) between the mutants and their parental strains are shown.

In order to make a more quantitative analysis of pH variation, an alternative liquid culture approach was used (Figure 4B,C) in a medium with very low glucose (0.2%). Under these conditions, the shift from fermentation to respiration can be followed before 24 h. Growth curves (Figure 4B) revealed that the T73 wild-type strain exhibited the fastest growth rate, followed by the *tsa1*Δ mutant, while the *trr1*Δ strain showed the slowest growth. These findings indicate that under low-glucose conditions, the deletion of cytosolic peroxiredoxin–thioredoxin reductase system components has a significantly greater impact than in glucose-rich environments (Figure 2A). Both T73 and *tsa1*Δ had a fast drop in pH during the fermentative phase, increasing later when cells are under respiration (Figure 4C). However, the pH increased in the wild-type strain more efficiently than in the *tsa1*Δ mutant, which maintained lower pH levels for longer times. The *TRR1* deletion strain exhibited delayed medium acidification and a slower subsequent rise in pH, reaching a level similar to that of the *tsa1*Δ mutant. Acetic acid was measured along the growth curve (Figure 4D). All strains showed an increase in acetic acid up to 8 h, which explains the drop in pH during fermentation. After that, acetic acid declined rapidly in the wild type but not so in the mutants, indicating that the thioredoxin–peroxiredoxin system impacts acetic acid assimilation in postdiauxic conditions.

On solid plates, the realkalization of the medium has been linked to ammonia production.^34^ When ammonia levels were measured in liquid cultures (Figure 4E), a later burst in ammonia production could contribute to the pH rise, but this event was not linked to either Trr1p or Tsa1p activity.

### Genetic Interaction between Tsa1p and Aldehyde Dehydrogenases

To understand how Tsa1p regulates acetate metabolism in wine yeasts, we explored its potential influence on primary aldehyde dehydrogenases. These enzymes are at the core of acetic acid production, with mitochondrial Ald4p primarily contributing during respiratory metabolism, while cytosolic Ald6p plays a dominant role under fermentative conditions. *ALD4* and *ALD6* deletion mutants were combined with *TSA1* knockout. To facilitate the construction of double mutants, the haploid wine strain C9 ^27^ was used in this section. First, the growth and acetate production of single and double mutants were tested in YPD plates containing the pH-sensitive dye BCP (Figure 5A). In this genetic background, the acid burst was more prolonged over time. *tsa1*Δ and *trr1*Δ mutants showed a lower level of acid production. As expected, the acidification was seriously impaired in the *ald4*Δ *s*train, confirming that Ald4p is the main enzyme involved in acetic acid production under these postdiauxic conditions. The deletion of *ALD6* also affected acid production, albeit to a lesser extent. The double mutant *tsa1*Δ*ald4*Δ *s*train displayed an acidification pattern similar to that of the *ald4*Δ single mutant but with a slight delay in medium realkalization. Interestingly, *tsa1*Δ*ald6*Δ showed evidence of an additive effect at longer times.

**Figure 5:**
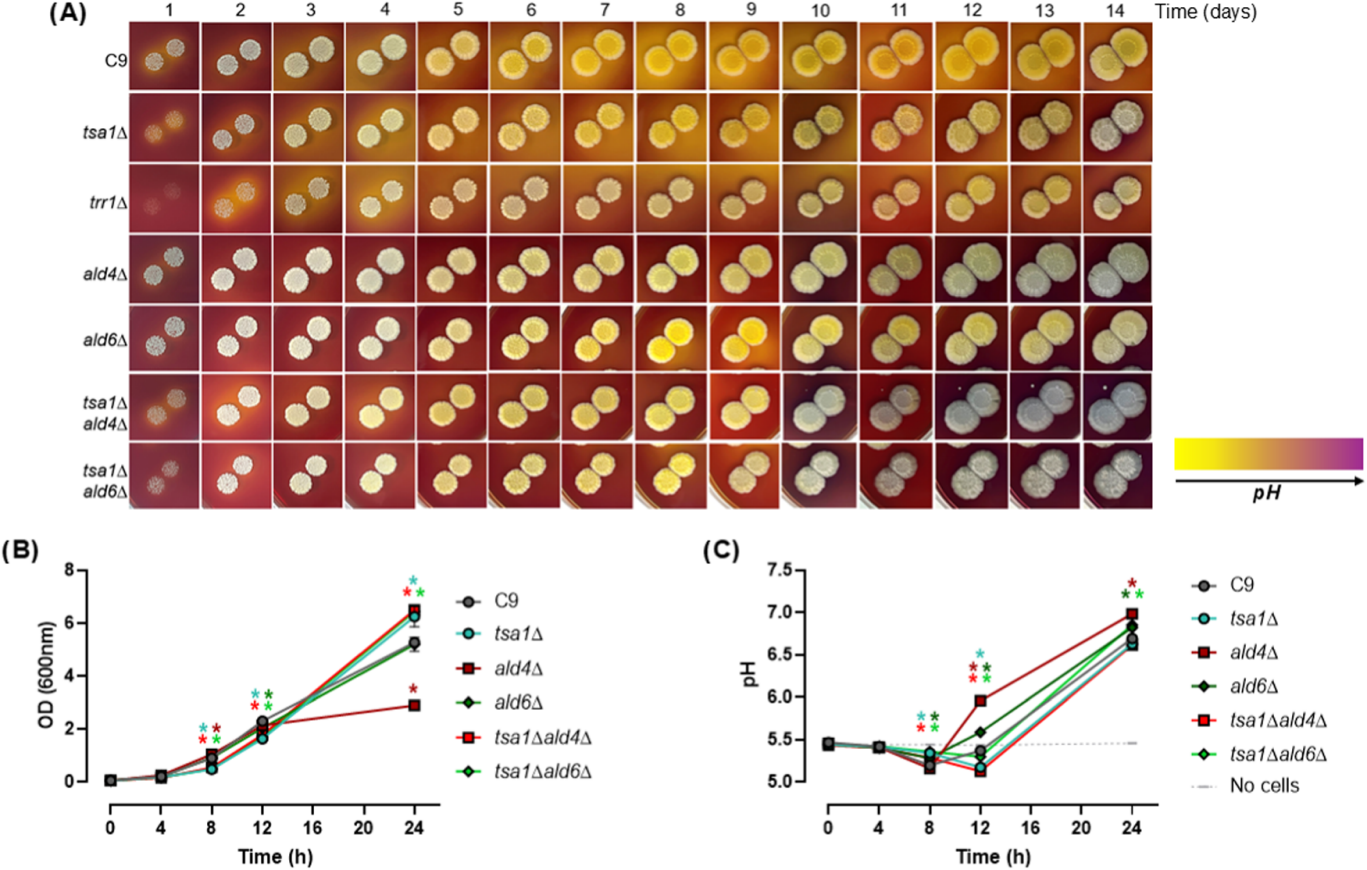
*TSA1* interacts genetically with *ALDH* in terms of medium acidification. Similar experiments of Figure 4 were performed with C9, *tsa1*Δ, *trr1*Δ̧ *ald4*Δ, *ald6*Δ, *tsa1*Δ*ald4*Δ, *a*nd *tsa1*Δ*ald6*Δ *s*trains. (A) Color evolution in YPD with BCP for 14 days. (B) Growth measured by OD_600_ in liquid medium with glucose 0.2%. (C) pH in the conditions described in (B). Experiments in liquid medium (B, C) were carried out in triplicate, and the average and standard deviation are provided. Significant differences (*p < 0.05, Student’s *t*-test) between the mutants and their parental strains are shown.

Growth under low carbon conditions can provide a clearer perspective of this interaction (Figure 5B,5C). Under these conditions, the growth profile of the *ald6*Δ strain was very similar to that of the parental strain (Figure 5B). In contrast, the *ald4*Δ strain grew similarly to the wild type during fermentative metabolism but reached lower densities at later stages when respiration is required. Therefore, Ald4p is relevant when mitochondrial metabolism is fully active. The *tsa1*Δ mutant exhibited slower growth than the wild type during the initial phase but reached a higher optical density at the end of the experiment. Interestingly, double mutants involving *tsa1*Δ displayed phenotypes that aligned closely with those of the *tsa1*Δ single mutant. Moreover, the growth defect of the *ald4*Δ mutant was completely suppressed by *TSA1* deletion, suggesting a compensatory interaction. The pH dynamics (Figure 5C) further clarified these relationships. As expected, the *ald4*Δ mutant exhibited the most pronounced impact, with a significant pH increase at later time points corresponding to respiratory growth. During early fermentative metabolism, acidification in the *ald4*Δ strain was unaffected. The *ald6*Δ mutant also influenced the pH increase but to a lesser extent. The *tsa1*Δ mutant displayed a delay in acidification during early growth but eventually reached nearly normal pH levels. Remarkably, *TSA1* deletion fully suppressed the pH phenotype of the *ald4*Δ mutant. In the *tsa1*Δ*ald6*Δ double mutant, the acidification delay observed in the *tsa1*Δ strain persisted, but the final levels were comparable to those of the *ald6*Δ mutant. This indicates a partial suppression effect, dependent on the metabolic status of the cell. After characterizing the genetic interactions between *TSA1* and *ALD4/6* in a standard laboratory medium, we extended our investigation to winemaking conditions. This fermentative and industrially relevant environment, where *S. cerevisiae* wine strains are typically employed, allowed us to assess the role of *TSA1* in regulating these enzymes under conditions that closely mimic actual wine production.

### Ald6p Activity is Controlled by Tsa1p during Winemaking

Peroxiredoxin Tsa1p exhibits a multifaceted role in acetic acid production in a variety of environments. However, the main biotechnological impact of acetic acid in winemaking lies in its contribution to volatile acidity during grape juice fermentation, so a closer look was taken at this process. In order to perform molecular analysis of vinification, a standard synthetic grape juice (MS300) was used (Figures 6 and 7) for microvinifications using T73 and T73 *tsa1*Δ strains. Fermentation was monitored by measuring reduced sugar consumption (Figure 6A). Fermentation speed was higher in the parental strain, which finished all sugars in 1 week, the expected behavior. However, the *tsa1*Δ mutant fermentation speed was very slow but ultimately completed fermentation to dryness in 26 days. Acetic acid production was measured during the 7 days that lasted the T73 fermentation (Figure 6B). In the wild-type strain, acetic acid was rapidly produced starting on day 1 and remained high during fermentation. In contrast, the *tsa1*Δ mutant displayed reduced acetic acid production on day 1, with levels recovering at later stages of fermentation. The current knowledge indicates that the major cytosolic aldehyde dehydrogenase Ald6p is the main contributor to acetic acid production during wine fermentation. Ald6p activity, measured in the presence of magnesium and NADP^+^ as a cofactor, supported this conclusion (Figure 6C). In parental T73, Ald6p activity peaked on the first day, corresponding to the rapid acetic acid production observed during this period. This activity subsequently decreased and stabilized at lower levels during the rest of the fermentation. *TSA1* deletion caused a decrease in activity on day 1, which matches the initial lower production of acetic acid (Figure 6B) and points to Tsa1p as a regulator of Ald6p activity. However, this activity in the mutant approached wild-type levels later on. To evaluate mitochondrial ALDH, its activity was measured in the presence of potassium ions and NAD^+^ as a cofactor (Figure 6D). As expected under fermentative conditions, Ald4p activity was substantially lower than that of Ald6p. In the T73 strain, it was negligible on day 1 when most acetic acid was produced but increased at longer times, albeit remaining significantly lower than the cytosolic activity. *TSA1* knockout did not notably impact this activity, as both wild-type and mutant strains showed similar patterns.

**Figure 6:**
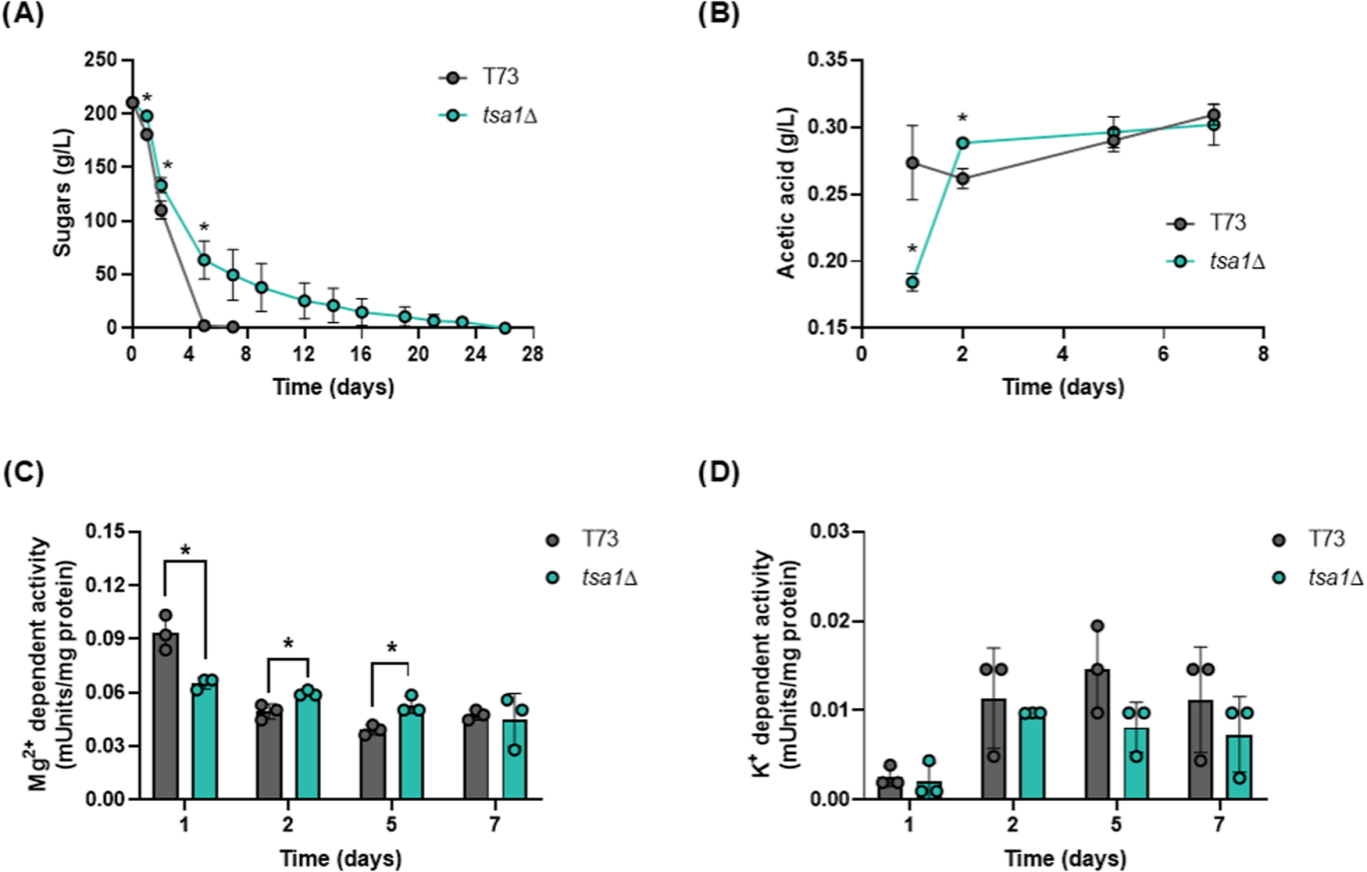
Tsa1p controls the Ald6p activity during winemaking. Fermentations on synthetic grape juice MS300 by T73 and T73 *tsa1*Δ strains were performed. (A) Fermentation evolution was measured as reducing sugar consumption. (B) Acetic acid production during the first 7 days of fermentation. (C) Cytosolic ALDH activity Ald6p during the time points described in panel (B). (D) Mitochondrial ALDH activity Ald4p during the time points described in panel (B). Experiments were carried out in triplicate, and the average and standard deviation are provided. Significant differences (*p < 0.05, Student’s *t-*test) between the mutant and the wild-type strains are shown.

**Figure 7:**
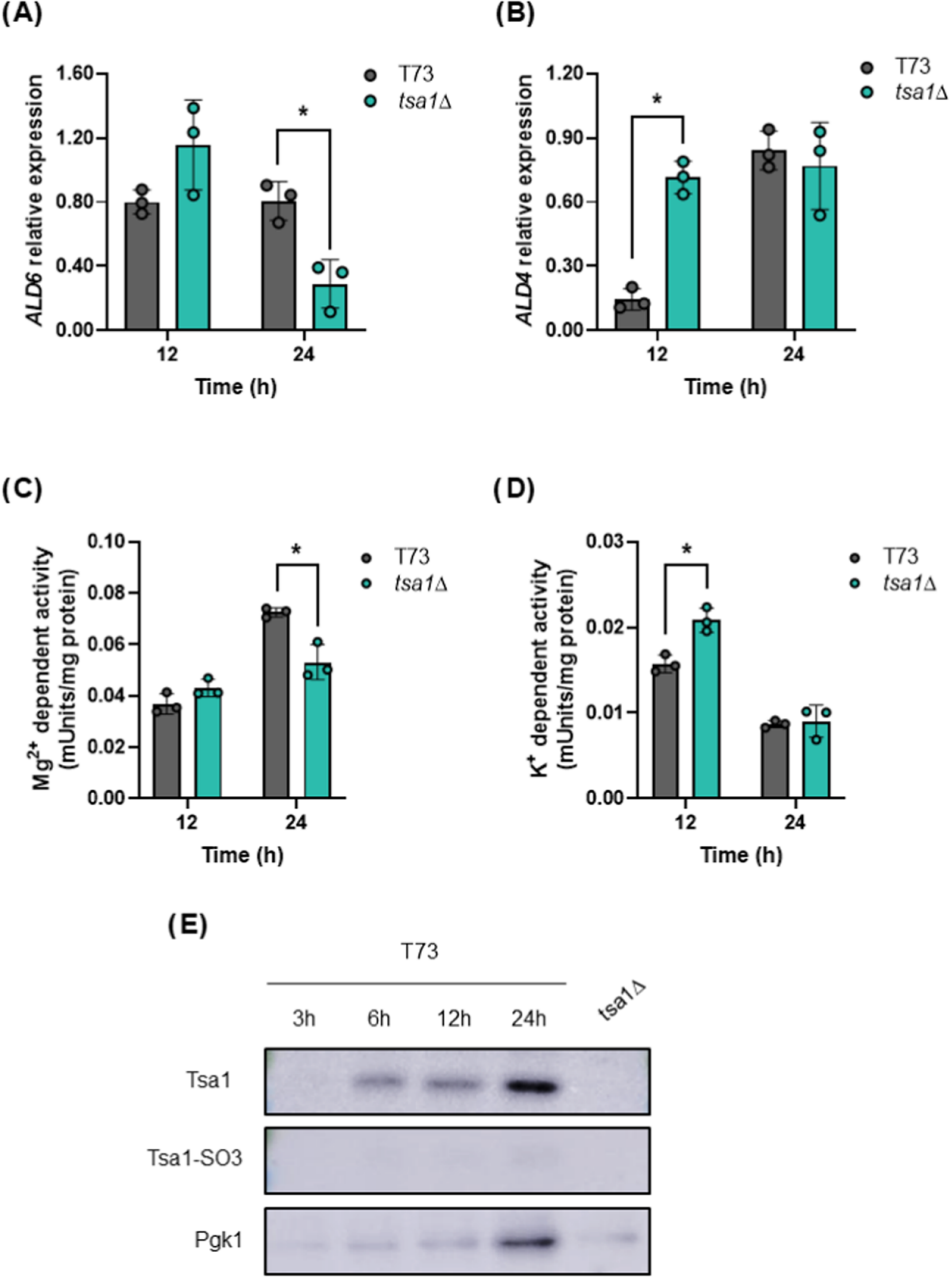
Tsa1p regulates early *ALD4/6* expression and activity. Fermentations on synthetic grape juice MS300 by T73 and T73 *tsa1*Δ strains were performed, and gene expression was measured in the first 24 h. (A) *ALD6* and (B) *ALD4* mRNA levels measured by qPCR. (C) Mg^2+^ dependent cytosolic ALDH activity at the same time points. (D) K^+^ dependent cytosolic ALDH activity at the same time points. (E) Western blot showing total and sulfenylated levels of Tsa1p in the wild-type T73 strain. A lane containing the protein extract from the T73 *tsa1*Δ strain at 24 h of fermentation was included as a negative control. Pgk1 levels were used as a loading control. Experiments were carried out in triplicate, and the average and standard deviation are provided. Significant differences (*p < 0.05, Student’s *t*-test) between the mutant and the wild-type strains are shown.

### Tsa1p Impacts Gene Expression

As the enzymatic activity of ALDH is affected by *TSA1* deletion, a closer look was taken at its potential mechanism. To do so, mRNA levels of the main aldehyde dehydrogenase genes *ALD6* and *ALD4*, along with their corresponding enzymatic activities, were analyzed at the beginning of the vinification, where the main differences were recorded (Figure 6). Quantitative PCR (qPCR) revealed distinct patterns of *ALD6* and *ALD4* expression over time (Figure 7A,B). For *ALD6*, mRNA levels were comparable between the wild-type and *tsa1*Δ strains at 12 h but were significantly reduced in the mutant at 24 h (Figure 7A). This aligns with the Mg^2+^-dependent cytosolic ALDH enzymatic activity (Figure 7C). *ALD4* gene expression exhibited a more complex profile. *TSA1* deletion increased *ALD4* levels at 12 h, but by 24 h, this difference was no longer observed (Figure 7B). Mitochondrial ALDH enzymatic activity mirrored this trend with elevated activity at 12 h in the mutant but no significant differences at 24 h between strains (Figure 7D). In this environment, *ALD4* is subject to glucose repression, which may mask any longer-term effects of *TSA1* deletion on mitochondrial ALDH activity. For this reason, the absolute levels of mitochondrial ALDH activity are lower than the cytosolic activity, causing only a small contribution to the final levels of acetate.

Finally, the levels of the Tsa1p protein itself during the first day of wine fermentation were measured by Western blot (Figure 7E). Tsa1p was barely detectable 3 h after inoculation but increased significantly over the next hours to reach a maximum at 24 h. The *tsa1*Δ strain was used as a control to confirm the antibody’s specificity. Oxidative stress was assessed by detecting hyperoxidized (sulfinylated) Tsa1p using a specific antibody. Tsa1p sulfinylated form indicates severe oxidative stress where the peroxiredoxin has been damaged and cannot be recycled by the redox-controlling machinery. No hyperoxidized Tsa1p was observed, suggesting the absence of oxidative damage during the initial stages of fermentation. This indicates that the regulatory role of Tsa1p in acetic acid metabolism and ALDH activity is not primarily driven by its antioxidant function.

## Discussion

The role of peroxiredoxin Tsa1p in acetic acid metabolism is described in detail in this work. The findings reveal a complex relationship as *TSA1* deletion impacts acetic acid production and consumption in diverse and sometimes apparently contradictory ways, depending on the growth medium and conditions. These conditions include both laboratory and industrially relevant settings, where exogenous pH is also significantly impacted.

Acetic acid is a critical compound in winemaking, as it constitutes the main chemical species behind undesirable volatile acidity. In previous work from our laboratory focused on the role of Tsa1p during biomass propagation, preliminary experiments during grape juice fermentation showed a decrease in acetic acid in the deletion mutant.^18^ Despite being a key issue in winemaking, acetic acid production and management during fermentation are not completely elucidated.^3^ Factors such as grape juice composition, particularly nitrogen and vitamin content, initial sugar concentration, and physical variables such as temperature and aeration all play significant roles in volatile acidity. To add more complexity to this issue, it is well-known that the genetic background strongly influences acetic acid production,^40–42^ so this is a factor taken into account when new strains are isolated from the wild and when the commercially available strains are marketed. We have observed variability in the impact of the deletion of peroxiredoxin *TSA1* and thioredoxin reductase *TRR1* on the growth on acetate as a carbon source and tolerance to high levels of acetic acid (Figure 2). These results suggest that redox control machinery may be contributing to the genetic heterogeneity observed in acetic acid metabolism and tolerance, an aspect that may warrant consideration in future studies and industrial applications. For the sake of experimental consistency, much of this work has focused on the commercial T73 strain. However, given the vast genetic variability among wine *S. cerevisiae* strains, conclusions drawn here may not apply universally across all strains.^43^

This study highlights that the *tsa1*Δ mutant impacts both the production and consumption of acetic acid. Even on the catabolic side of metabolism, the effect of *TSA1* deletion is highly dependent on the initial metabolic conditions. For instance, the *tsa1*Δ deletion mutant shows accelerated acetic acid consumption during the postdiauxic phase of a 1% glucose culture (Figure 3) but exhibits elevated acetic acid levels when the culture is mainly respiratory, with just 0.2% glucose (Figure 4D). This underscores the role of the thioredoxin–peroxiredoxin system in sensing and adapting the cellular metabolic status to regulate metabolism. While this study primarily focuses on enzymes responsible for acetic acid production, the catabolic side of metabolism is also crucial. When acetate is the sole carbon and energy source, it is metabolized to acetyl-CoA by acetyl-CoA synthase enzymes. As *tsa1*Δ mutants display impaired growth on acetate (Figure 2A), and since Tsa1p is a cytosolic protein, it may exert its regulatory influence over the Acs1 isoform, which mainly provides cytosolic acetyl-CoA for lipid biosynthesis. We have previously observed an impact of thioredoxin deletion on lipid biosynthesis that may point in the same direction.^19^ A deficiency in lipid synthesis could partially explain the poor growth of the mutant under these conditions. Furthermore, acetyl-CoA generated by these enzymes is required not only for lipid synthesis but also for chromatin silencing. This dual role could suggest that alterations in acetyl-CoA metabolism may indirectly affect gene expression, potentially contributing to the range of phenotypes observed in the *tsa1*Δ mutant.

Production of acetic acid under fermentative conditions primarily arises from the oxidation of part of the acetaldehyde produced as an intermediate in alcoholic fermentation by a variety of aldehyde dehydrogenases. Under winemaking conditions, the main cytosolic ALDH (Ald6p) is the predominant contributor to acetic acid production, while the mitochondrial isoform (Ald4p) is less active.^4,7^ This was confirmed in our experiments (Figure 6), where the reduction in acetic acid levels in the *tsa1*Δ mutant was correlated with a decrease in the magnesium- and NADP^+^-dependent ALDH activity linked to the cytosolic Ald6p isoenzyme. The genetic interaction analysis revealed a more intricate regulatory picture (Figure 5). Interestingly, the absence of *TSA1* suppressed the phenotype of both *ALD4* and *ALD6* deletion mutants. Individually, these mutants increased the pH due to lower acetic acid production under respiratory laboratory conditions. The observed alkalinization linked to ammonia secretion has been associated with cellular communication between cells inside a colony.^34^ However, our data show that Tsa1p had no direct effect on ammonia production. Instead, its impact was linked to the acid burst associated with acetic acid. This phenomenon, previously connected to mitochondrial superoxide dismutase (Sod2) through Ald4p,^33^ has now been shown to also involve peroxiredoxins under our respiratory conditions, placing Tsa1p as a key regulatory node. Our previous work shows that cytosolic thioredoxin mutant regulates cytosolic Sod1 as well.^44^ Superoxide dismutases (SODs) transform superoxide into hydrogen peroxide that can act as a second messenger in different processes.^45^ Tsa1p may be the sensor of some signals generated by SODs to control both aldehyde dehydrogenases.

Peroxiredoxins, particularly Tsa1p, are increasingly recognized as regulators of cellular processes rather than merely detoxifying enzymes. Evidence from this study supports this perspective, as the absence of hyperoxidation in Tsa1p catalytic cysteine during winemaking (Figure 7) indicates that its regulatory role is triggered by more subtle inputs than a stressful rise in reactive oxygen species (ROS). The mechanism of action of Tsa1p can be either direct or indirect. It is known that peroxiredoxins modulate the activity of enzymes and transcription factors by regenerating oxidized cysteine residues. For instance, the oxidative-responsive transcription factor Yap1 is activated via the formation of a disulfide bond, which increases its nuclear localization. This redox regulation has been linked to the ability of peroxiredoxin to relay their oxidative status.^46^ Mutations in *YAP1* have previously been associated with altered acetic acid production during winemaking,^47^ suggesting that Tsa1p may regulate gene expression involved in acetic acid metabolism through a similar mechanism. Specifically, Tsa1p might directly regulate the redox status of cysteine residues of one or several enzymes involved in acetic acid synthesis and/or degradation. Among them, enzymes sharing cytosolic localization with Tsa1p are the most likely candidates, with aldehyde dehydrogenase Ald6p being a prominent example. Global analyses suggest that Tsa1p may bind to Ald6p under specific conditions and that Ald6p contains a cysteine residue susceptible to redox regulation.^48^ However, direct regulation alone cannot account for the modulation of mitochondrial ALDH Ald4p, making an indirect mechanism that also encompasses transcriptional regulation the most reasonable explanation. A likely candidate for this mechanism is the ability of Tsa1p to modulate the nutrient signaling pathway through protein kinase A (PKA).^23,49^ The cAMP-dependent PKA pathway promotes growth and protein synthesis under glucose-rich conditions. Tsa1p has been shown to stimulate the sulfenylation of cysteine residues in the PKA catalytic subunit, thereby repressing it. PKA activity regulates numerous metabolic pathways and represses the general stress response, acting at both the transcriptional and post-transcriptional levels. This multifaceted regulation provides several potential ways to control the acetic acid metabolism. For instance, in previous work, we described that *ALD4* was regulated by the general stress response transcription factors Msn2/4, direct targets of PKA.^6^ The net impact of *TSA1* deletion on acetic acid levels is highly dependent on the growth and metabolic status of the cell, leading to seemingly contradictory outcomes. One potential explanation for this erratic behavior may lay in the fact that the oxidation cycles of peroxiredoxins, including Tsa1p, have been linked to metabolic oscillations.^50^ Hence, the absence of Tsa1p might exert opposite effects in different phases of these circadian rhythms, such as yeast respiratory oscillation. Therefore, there is still much work ahead to fully grasp the nuances of how the redox regulatory machinery influences metabolism. Addressing this challenge will not only improve our understanding of these processes but also provide innovative strategies to modulate the production of relevant molecules during industrial yeast fermentations.

## Supplementary Material



## Notes

PDH bypass scheme (Figure S1); influence of Tsa1p in acetic acid production and consumption in laboratory growth media in a laboratory yeast strain (Figure S2); list of the strains used in this study (Table S1); absolute values of glucose, ethanol, acetic acid, and growth (OD_600_) of T73 and T73 tsa1Δ strains during growth in GMM (Table S2) (PDF)
