# Supplementary material for: Cytosolic Peroxiredoxin TSA1 Influences Acetic Acid Metabolism and pH Homeostasis in Wine Yeasts
Source: J Agric Food Chem. 2025 Mar 22;73(13):8015–25. doi: 10.1021/acs.jafc.4c13199 (PMC12510144; doi:10.1021/acs.jafc.4c13199)
Supplement: Supplementary file 1 [file jf4c13199_si_001.pdf]

Supplementary Material to "Cytosolic peroxiredoxin *TSA1* influences acetic acid metabolism and pH homeostasis in wine yeasts" by Víctor Garrigós[1], Cecilia Picazo[1], Lisa Dengler[2], Jennifer C. Ewald[2], Emilia Matallana[1], Agustín Aranda[1]*. It contains Supplementary Figure 1, Supplementary Figure 2, Supplementary Table 1 and Supplementary Table 2.

[1] Institute for Integrative Systems Biology (I2SysBio), Universitat de València-CSIC, C/ Catedrático Agustín Escardino 9, 46980, Paterna, Valencia, Spain

[2] Interfaculty Institute of Cell Biology (IZB), University of Tuebingen, Auf der Morgenstelle 15, 72076, Tuebingen, Germany

* Email: agustin.aranda@csic.es

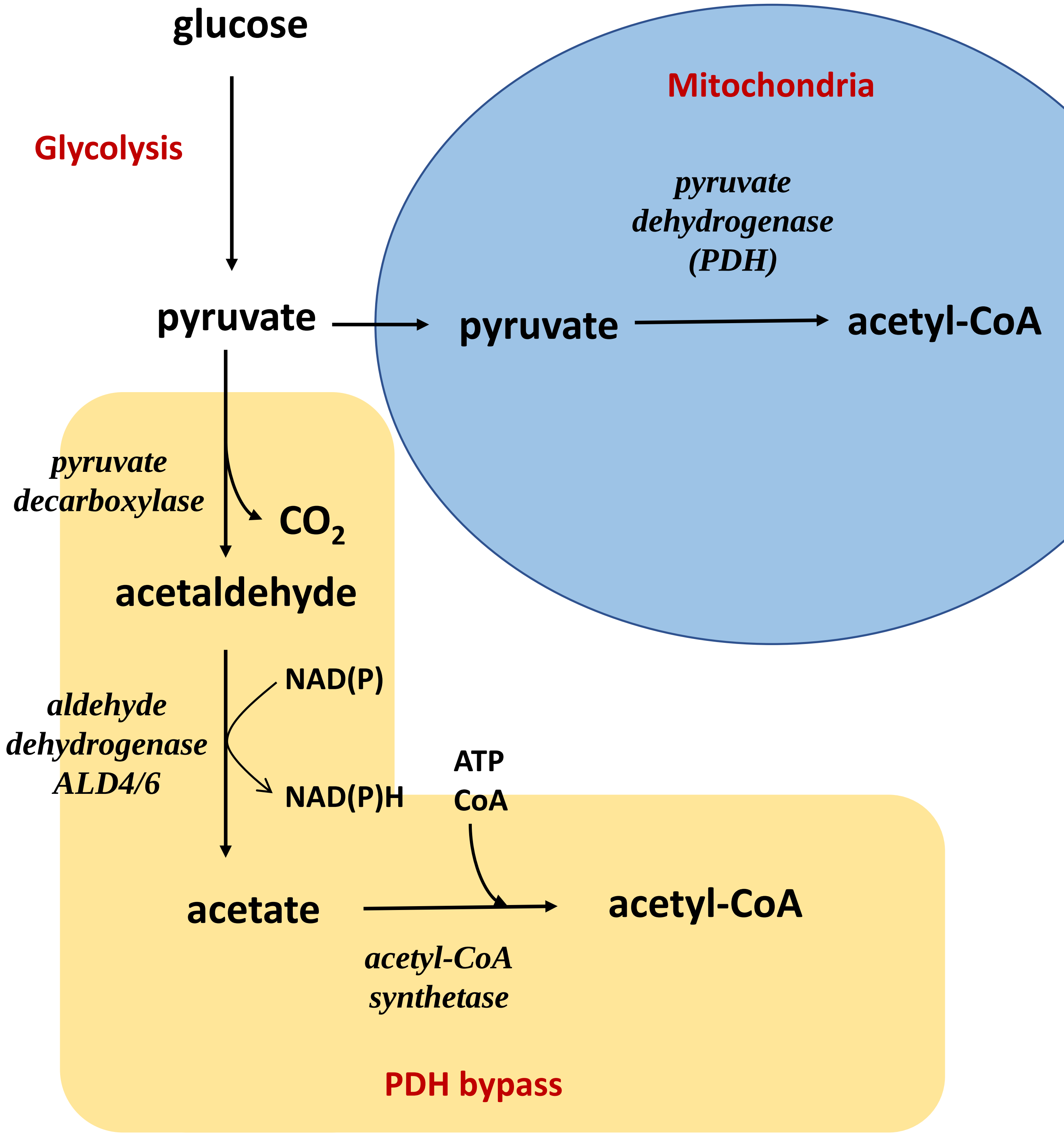

Supplementary Figure 1. PDH bypass scheme.
Enzymes are indicated in italics and metabolites in plain text.
Blue: mitochondria. Orange: PDH bypass reactions

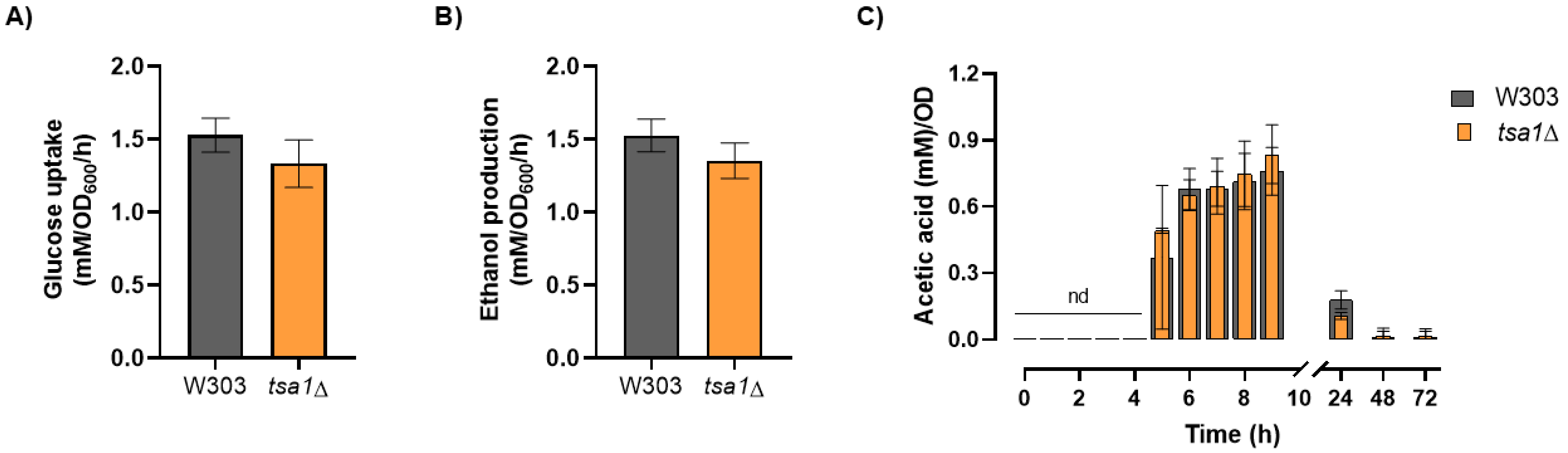

Supplementary Figure 2. Influence of Tsa1p in acetic acid production and consumption in laboratory growth medium in a laboratory yeast strain. A) Glucose uptake B) ethanol production and C) Acetic acid yields in GMM medium by W303 and W303 *tsa1Δ* strains. Experiments were carried out in triplicate, and the average and standard deviation are provided. Significant differences (*$p < 0.05$, Student's t-test) between the mutants and their parental strains are shown.

Supplementary Table 1. List of the strains used in this study.

Yeast Strain	Genotype / Plasmid added	Source
L2056	Commercial Wine Strain Lalvin Rhône 2056™	Lallemand Inc.
L2056 *tsa1Δ*	L2056 *tsa1*::*loxP tsa1*::*KanMX*	Garrigós et al., 2020
L2056 *trr1Δ/TRR1*	L2056 *trr1*::*KanMX TRR1*	Garrigós et al., 2020
L2056 *tsa1Δ*	L2056 CRISPR/Cas9 *tsa1Δ*	This study
L2056 *trr1Δ*	L2056 CRISPR/Cas9 *trr1Δ*	This study
T73	Commercial Wine Strain Lalvin T73™	Lallemand Inc.
T73 *tsa1Δ*	T73 CRISPR/Cas9 *tsa1Δ*	This study
T73 *trr1Δ*	T73 CRISPR/Cas9 *trr1Δ*	This study
EC1118	Commercial Wine Strain Lalvin EC1118™	Lallemand Inc.
EC1118 *tsa1Δ*	EC1118 CRISPR/Cas9 *tsa1Δ*	This study
EC1118 *trr1Δ*	EC1118 CRISPR/Cas9 *trr1Δ*	This study
W303	MAT a, *ADE*, *LEU*, *HIS*, *TRP*, *URA*	Jennifer C. Ewald laboratory
W303 *tsa1Δ*	W303 *tsa1*::*loxP*	This study
C9	C9 Mat a *ho*::loxP	Walker et al., 2003
C9 *tsa1Δ*	C9 *tsa1*::*KanMX*	Picazo et al., 2018
C9 *ald4Δ*	C9 *ald4*::*KanMX*	H. Orozco
C9 *ald6Δ*	C9 *ald6*::*KanMX*	H. Orozco
C9 *tsa1Δ ald4Δ*	C9 *tsa1*::*loxP ald4*::*KanMX*	This study
C9 *tsa1Δ ald6Δ*	C9 *tsa1*::*loxP ald6*::*KanMX*	This study

Supplementary Table 2. Absolute values of glucose, ethanol, acetic acid and growth (OD600) of T73 and T73 *tsa1*Δ strains during growth in GMM. Experiments were carried out in triplicate, and average and standard deviations are provided.

	T73				*tsa1*Δ			
Time (h)	Glucose (mM)	Ethanol (mM)	Acetic acid (mM)	OD (600nm)	Glucose (mM)	Ethanol (mM)	Acetic acid (mM)	OD (600nm)
0	50.94 ± 0.70	3.58 ± 0.15	nd	0.20 ± 0.00	51.04 ± 0.61	3.20 ± 0.70	nd	0.20 ± 0.00
1	50.03 ± 0.67	4.86 ± 0.07	nd	0.31 ± 0.01	50.01 ± 0.71	4.85 ± 0.73	nd	0.34 ± 0.06
2	48.70 ± 0.55	6.83 ± 0.53	nd	0.41 ± 0.01	48.54 ± 0.64	7.04 ± 0.68	nd	0.43 ± 0.06
3	46.09 ± 0.55	11.02 ± 0.24	nd	0.64 ± 0.02	46.22 ± 1.40	10.42 ± 1.80	nd	0.65 ± 0.09
4	41.28 ± 0.93	18.19 ± 0.37	nd	1.04 ± 0.11	42.19 ± 1.96	16.34 ± 2.57	nd	0.99 ± 0.11
5	34.63 ± 1.70	28.57 ± 1.56	1.06 ± 0.11	1.37 ± 0.09	37.16 ± 3.15	24.02 ± 3.99	0.96 ± 0.20	1.28 ± 0.16
6	24.57 ± 0.69	43.71 ± 1.98	1.88 ± 0.15	2.21 ± 0.31	29.96 ± 2.39	34.68 ± 3.00	1.44 ± 0.15	1.98 ± 0.37
7	12.74 ± 2.39	61.86 ± 4.15	3.16 ± 0.49	3.22 ± 0.29	21.01 ± 4.13	48.28 ± 5.67	2.06 ± 0.35	2.74 ± 0.37
8	1.44 ± 0.77	78.53 ± 1.23	4.45 ± 0.36	4.33 ± 0.06	10.96 ± 4.29	63.19 ± 5.15	2.79 ± 0.38	3.67 ± 0.15
9	0.01 ± 0.01	79.26 ± 2.72	5.03 ± 0.36	4.80 ± 0.10	3.62 ± 3.03	73.54 ± 1.69	3.46 ± 0.46	4.23 ± 0.15
24	0.00 ± 0.00	33.95 ± 1.48	5.25 ± 1.13	6.93 ± 0.12	0.00 ± 0.00	44.24 ± 1.29	1.44 ± 0.77	6.10 ± 0.17
48	0.00 ± 0.00	0.00 ± 0.00	0.81 ± 0.04	8.13 ± 0.42	0.00 ± 0.00	0.00 ± 0.00	0.30 ± 0.26	7.60 ± 0.35
72	0.00 ± 0.00	0.00 ± 0.00	0.78 ± 0.09	8.40 ± 0.35	0.00 ± 0.00	0.00 ± 0.00	0.13 ± 0.23	7.80 ± 0.35
